# The dual role of urinary C-peptide/creatinine ratio: predicting insulin resistance in non-diabetic adults and microvascular complications risk in patients with type 2 diabetes

**DOI:** 10.3389/fendo.2026.1786731

**Published:** 2026-03-19

**Authors:** Shufen Yin, Ling Zhong, Lanyu Gao, Qing Shao, Liu Wang, Yuwei Zhang

**Affiliations:** 1 Department of Endocrinology and Metabolism, West China Hospital, Sichuan University, Chengdu, China; 2 Center for Diabetes and Metabolism Research, West China Hospital of Sichuan University, Chengdu, China

**Keywords:** fasting urinary C-peptide/creatinine ratio, 2-hour postprandial urinary C-peptide/creatinine ratio, insulin resistance, diabetic microvascular complications, predictive value

## Abstract

**Background and purpose:**

Insulin resistance (IR) is a key driver of type 2 diabetes mellitus (T2DM) onset, and diabetic microvascular complications (DMC) represent its poor prognosis. However, simple and non-invasive screening methods for detection of IR and DMC are limited. The urinary C-peptide/creatinine ratio (UCPCR) can be used to measure β-cell function, and we aimed to evaluate its utility as a surrogate marker for IR in non-diabetic individuals and for DMC risk in patients with T2DM.

**Methods:**

This cross-sectional study enrolled 447 individuals (255 non-diabetic adults and 192 T2DM patients) from November 2023 to September 2025. The non-diabetic cohort was divided into IR and non-IR groups according to the Matsuda index. The diabetic cohort was divided into DMC and non-DMC groups. Fasting UCPCR (0hUCPCR) and 2-h post-OGTT/steamed bread meal (2hUCPCR) were measured. Multiple regression analysis was used to describe the association between UCPCR and the Matsuda index or DMC. Diagnostic accuracy was assessed using receiver operating characteristic (ROC) curve analysis.

**Results:**

In the non-diabetic individuals, both 0hUCPCR and 2hUCPCR were higher in the IR group, and they were negatively associated with the Matsuda index (*r* = −0.488, *r* = −0.636) (all *P* < 0.001) by Spearman’s correlation analysis. Multiple linear regression analysis demonstrated that 0hUCPCR (*B* = −0.351) and 2hUCPCR (*B* = −0.162) were negatively associated with the Matsuda index (all *P* < 0.001). ROC analysis revealed that the areas under the curve (AUC) for screening IR were 0.780 and 0.831, with corresponding sensitivities of 64.4% and 77.6%, respectively. In the T2DM patients, 0hUCPCR and 2hUCPCR were lower in the DMC group, and they showed positive correlations with HOMA-β (*r* = 0.582, *r* = 0.617) (all *P* < 0.001). Multivariable logistic regression analysis demonstrated that 2hUCPCR was negatively associated with DMC (OR = 0.627, *P* = 0.030). ROC analysis revealed that 2hUCPCR predicted DMC risk with an AUC of 0.751 and a sensitivity of 70.7%.

**Conclusion:**

Both 0hUCPCR and 2hUCPCR are promising markers for IR in non-diabetic adults, and 2hUCPCR is associated with lower risk of DMC in T2DM patients. Coupled with their practical advantages of simplicity and non-invasiveness, UCPCRs are promising tools for future large-scale screening in at-risk populations.

## 1 Introduction

According to the International Diabetes Federation, diabetes affected 11.11% of the global adult population, corresponding to 589 million adults in 2024, and is projected to affect 12.96% and 853 million people aged 20–79 years by 2050. China bears the largest share of this burden, with 148 million people affected (1). Type 2 diabetes mellitus (T2DM) accounts for over 90% of diabetes cases and represents a major threat to patient health and survival (2). T2DM is characterized by a non-autoimmune heterogeneously progressive loss of adequate islet β-cell insulin secretion. This process is frequently accompanied by insulin resistance (IR), a state defined by reduced tissue sensitivity and responsiveness to insulin (3). Notably, IR often develops insidiously and usually occurs several years or even decades before the onset of diabetes (4). Research indicated that IR can already be present in non-obese individuals with normal fasting plasma glucose (FPG) and is associated with higher risk of cardiometabolic diseases (5, 6). Therefore, implementing early screening for IR is critical for diabetes prevention. The hyperinsulinemic–euglycemic clamp technique is regarded as the gold standard for IR measurement, yet it is complicated, time-consuming, and costly (7). In clinical practice, IR is commonly assessed using blood insulin or the homeostasis model assessment of insulin resistance (HOMA-IR). However, these indicators merely reflect basal insulin secretion levels and fail to adequately capture dynamic insulin secretion or whole−body insulin sensitivity. Moreover, their labor−intensive nature makes them unsuitable for epidemiological studies and large−scale clinical investigations (8).

As T2DM progresses to its advanced stages, it is marked by a deterioration of β-cell function, which is closely linked to the development of diabetic microvascular complications (DMC). β-cell dysfunction directly impairs glycemic control, resulting in glucose variability and chronic hyperglycemia that are critical risk factors for DMC (9, 10). Among over 6 million T2DM inpatients in Chinese tertiary hospitals, the prevalence of diabetic microvascular complications (DMC) increased from 19.0% in 2013 to 21.0% in 2017. DMC primarily comprises diabetic kidney disease (DKD), diabetic retinopathy (DR), and diabetic peripheral neuropathy (DPN) (11). These complications are leading causes of end-stage renal failure, blindness, disability, and survival loss, profoundly reducing both life expectancy and the quality of life (12, 13). Currently, DMC are evaluated using techniques such as fundus examinations, urinary albumin measurements, and neurological function assessments (14). However, these methods require specialized equipment and complex procedures, limiting their feasibility for large-scale screening. Blood C-peptide and homeostasis model assessment of β-cell function (HOMA-β) are commonly used to assess β-cell dysfunction (15). However, exogenous insulin confounds measurements based on circulating insulin in patients receiving insulin therapy (15). Additionally, C-peptide is limited by its transient secretion, poor stability *in vitro*, and the requirement for invasive blood collection (16).

5%-10% of serum C-peptide is excreted renally as urinary C-peptide (UCP). UCP provides an integrated measure of C-peptide secretion over several hours. It remains stable at room temperature for 24 h without preservatives and can be obtained non-invasively (17). The urinary C-peptide/creatinine ratio (UCPCR) standardizes the UCP concentration by creatinine, thereby compensating for variations in urine concentration and offering a more reliable assessment. In addition, UCPCR is less influenced by renal function compared with UCP (18). Prior evidence has shown that fasting UCPCR positively correlates with HOMA-IR and can predict prediabetes in apparently healthy adults (19). Hassan et al. reported that a postprandial UCPCR ≥ 0.20 nmol/mmol following a mixed meal can diagnose IR in children with obesity (20). However, no study to date has examined the utility of UCPCR for IR screening among adults. In patients with diabetes, studies indicated that fasting and postprandial UCPCR are indicative of endogenous insulin production and show promise for diabetes classification (21–23). Whether UCPCR is associated with DMC risk and its predictive value in this context remain unclear. Meanwhile, comparisons between fasting and postprandial UCPCR have been limited. Therefore, this study aimed to investigate the utility of fasting and 2−h post−load UCPCR (0hUCPCR, 2hUCPCR) for screening IR in non−diabetic adults and predicting DMC in patients with T2DM.

## 2 Materials and methods

### 2.1 Participants

This prospective cross-sectional study enrolled adults (≥18 years old), comprising patients with T2DM and individuals without diabetes. Participants were recruited from the inpatient ward (T2DM group) and the outpatient clinic (non-diabetic group) of the Department of Endocrinology and Metabolism, West China Hospital, Sichuan University, between November 2023 and September 2025. The diagnosis of normal glucose tolerance (NGT), prediabetes (Pre-DM), and diabetes followed the criteria set by the American Diabetes Association in 2024 (24). Exclusion criteria were as follows: (1) acute diabetic complications (e.g., ketoacidosis and hyperosmolar hyperglycemic state), fasting plasma glucose ≥ 10 mmol/L on the day of urine collection; (2) severe cardiac or hepatic insufficiency; (3) estimated glomerular filtration rate (eGFR) < 45 mL/min/1.73 m^2^; (4) malignancy; (5) acute infection or inflammatory conditions; (6) major psychiatric disorders; (7) pregnancy or lactation; (8) current use of angiotensin receptor-neprilysin inhibitors (25). The study was conducted in accordance with the Declaration of Helsinki and was approved by the Ethics Committee of West China Hospital, Sichuan University (No. 1179–2021 Audit). Written informed consent was obtained from all participants. After a 10-12-h overnight fast, patients with T2DM were given a 100-g steamed bread and non-diabetic individuals underwent a 75-g oral glucose tolerance test (75 g OGTT). Venous blood was collected at fasting, 1 h, and 2 h postprandially. Urine samples included the second voided fasting (after discarding the first morning urine) and a pooled 2-h postprandial collection. All urine samples were centrifuged at 3,500 rpm for 20 min at 4 °C, and the supernatant was aliquoted and stored at −80 °C until batch analysis.

### 2.2 Diagnostic criteria

In the non-diabetic individuals, IR was defined as a Matsuda index < 4.3 (26). The Matsuda index is an index of whole-body insulin sensitivity derived from the simultaneous assessment of insulin and glucose levels during an OGTT. It considers insulin sensitivity in the basal state and after the ingestion of a defined glucose load, thus offering a more accurate assessment (7). In the T2DM patients, DMC were diagnosed according to the Chinese Guidelines for the Prevention and Treatment of Type 2 Diabetes (2020 Edition) (27). Specifically, DKD was diagnosed based on a urinary albumin/creatinine ratio ≥ 30 mg/g on two separate tests or a sustained eGFR < 60 mL/min/1.73 m² for over 3 months, having ruled out other renal pathologies. DR was identified by the presence of microaneurysms, hemorrhages, or other definitive diabetic lesions on fundus examination. DPN required both neuropathic symptoms (e.g., numbness and pain) and at least one objective sign from standardized tests assessing pressure, vibration, temperature, ankle reflexes, or pinprick sensation. Patients with one or more of these criteria were classified into the DMC group, whereas those meeting none were assigned to the non-DMC group.

### 2.3 Baseline data and laboratory measurements

Baseline data were collected including (1) demographic and clinical characteristics: sex, age, smoking status, comorbidities, and current medications; (2) anthropometric measures: height, weight, waist circumference, body mass index (BMI), and waist-to-height ratio (WHtR); (3) disease-specific metrics: diabetes duration, systolic blood pressure (SBP), diastolic blood pressure (DBP); and (4) key laboratory parameters: plasma glucose, triglycerides (TG), total cholesterol (TC), high-density lipoprotein cholesterol (HDL-C), low-density lipoprotein cholesterol (LDL-C), eGFR, uric acid (UA), and urinary creatinine were analyzed using a Roche Cobas C702 biochemical analyzer. Glycated hemoglobin (HbA1c) was assessed with ARKRAY HA-8190V (ADAMS) hemoglobin analyzer. Serum C-peptide and insulin were measured using a Roche Cobas 8000 e801 electrochemiluminescence immunoassay analyzer. Urinary C-peptide was measured using the DRG Human C-peptide ELISA kit (Germany), with a measurement range of 0–16 ng/mL and intra- and interassay coefficients of variation of <3.1% and <4.3%, respectively.

### 2.4 Calculations

The BMI was calculated as weight (kg)/height^2^(m)^2^ and WHtR as waist (cm)/height (cm). HOMA-IR was calculated using the following formula: fasting insulin (μU/mL) × fasting plasma glucose (mmol/L)/22.5; HOMA-β was calculated using the following formula: 0.27 × fasting C-peptide (pmol/L)/[fasting plasma glucose (mmol/L)-3.5]; and the Matsuda index was calculated as 10,000/√[fasting glucose (mg/dL) × fasting insulin (μU/mL) × mean glucose (mg/dL) × mean insulin (μU/mL) during OGTT], where mean glucose and insulin were the averages of the 0-, 60-, and 120-minute OGTT values. Triglycerides and glucose (TyG) index = Ln (FPG mg/dL × triglycerides (mg/dL))/2, TyG-body mass index (TyG-BMI) = Ln (FPG mg/dL × triglycerides (mg/dL))/2 × BMI, lipid accumulation product (LAP) = (WC − 65) × triglycerides (mmol/L) for men, or (WC − 58) × triglycerides (mmol/L) for women.

### 2.5 Statistical analysis

Data were analyzed by SPSS software (IBM SPSS 25.0). For normally distributed continuous variables, data are presented as mean ± SD and compared with the independent t-test between the two groups. For non-normally distributed variables, data are presented as median (IQR) and compared with the Mann–Whitney U test. Categorical variables were presented as n (%) and compared using the chi-square test. Spearman’s correlation analysis was performed to evaluate associations between UCPCRs and IR/β-cell function indices. Multiple linear regression analysis was performed to assess the independent association between UCPCRs and the Matsuda index. Using the natural logarithm of the Matsuda index as the dependent variable, 0hUCPCR and 2hUCPCR were included as independent variables of interest in separate models. The unstandardized coefficient *B* and its 95% confidence interval ((95% CI) represent the statistical effect. Binary logistic regression analysis was used to determine association between UCPCRs and DMC. Using DMC (0 = no, 1 = yes) as dependent variable, two separate models were constructed for 0hUCPCR and 2hUCPCR. The odds ratio (OR) and its 95% CI were used to estimate the statistical effect. Covariates for the multivariate models were adjusted sequentially for demographic factors, variables with *P* ≤ 0.1 in the univariate analysis after excluding multicollinear variables. ROC curve analysis was used to determine the optimal UCPCR cutoff for screening IR in non-diabetic individuals and predicting DMC risk in T2DM patients. The AUC serves as a metric for predictive performance. A two-tailed *P* < 0.05 was considered statistically significant. Separate models were built for each individual complication (DKD, DR, and DPN). To address the issue of multiple testing, a Bonferroni correction was applied to these three secondary analyses, and statistical significance was defined as *P* < 0.017 (0.05/3).

## 3 Results

### 3.1 Baseline characteristics of the study participants

Among the 255 non-diabetic participants, 103 (40.4%) were male; the median age was 35.0 years (IQR 25.0-43.0). Based on IR status, they were categorized into an IR group (n=205) and a non-IR group (n=50). **Table 1** shows that the IR group, compared with the non-IR group, had a higher proportion of men and elevated BMI, WHtR, eGFR, UA, TG, HbA1c, FPG, 1-h plasma glucose (1hPG), 2hPG, fasting insulin (FINS), 1-h insulin (1hINS), 2-h insulin (2hINS), HOMA-IR, 0hUCPCR, and 2hUCPCR (all *P* < 0.05) alongside a lower age, HDL-C, and Matsuda index (all *P* < 0.05). Other clinical parameters showed no significant intergroup differences (all *P* > 0.05).

**Table 1:** The baseline clinical characteristics of non-diabetic adults.

Variable	Overall (n = 255)	Non-IR (n = 50)	IR (n = 205)	*P* value
Male, n (%)	103 (40.4)	13 (26.0)	90 (43.9)	**0.021**
Age (years)	35.0 (25.0, 43.0)	42.5(37.0, 54.0)	32.0 (23.0, 40.5)	**< 0.001**
Smoker, n (%)	25 (9.8)	3 (6.0)	22 (10.7)	0.313
BMI (kg/m^2^)	28.70 (24.24, 33.67)	22.47 (20.83, 26.15)	30.00 (26.51, 34.76)	**< 0.001**
WHtR	0.58 (0.52, 0.63)	0.50 (0.48, 0.54)	0.60 (0.54, 0.64)	**< 0.001**
SBP (mmHg)	122 ± 12	119 ± 15	122 ± 11	0.086
DBP (mmHg)	76 ± 9	76 ± 7	77 ± 9	0.669
eGFR (ml/min/1.73m^2^)	108.59 ± 15.11	103.83 ± 11.36	109.87 ± 15.75	**0.009**
UA (µmol/L)^*^	382 (294, 450)	251 (227, 363)	398 (330, 473)	**< 0.001**
TG (mmol/L)^*^	1.43 (1.07, 2.23)	1.17 (0.87, 1.67)	1.52 (1.10, 2.39)	**0.006**
TC (mmol/L)^*^	4.66 ± 0.96	4.50 ± 0.91	4.70 ± 0.96	0.271
HDL-C (mmol/L)^*^	1.09 (0.97, 1.31)	1.19 (1.02, 1.52)	1.07 (0.97, 1.27)	**0.029**
LDL-C (mmol/L)^*^	2.92 ± 0.86	2.75 ± 0.91	2.96 ± 0.84	0.196
HbA_1c_ (%)^*^	5.6 (5.4, 5.9)	5.4 (5.2, 5.7)	5.6 (5.4, 5.9)	**0.001**
FPG (mmol/L)	5.29 (4.93, 5.66)	4.94 (4.73, 5.33)	5.40 (5.01, 5.71)	**< 0.001**
1hPG (mmol/L)	9.60 (7.97, 10.96)	8.22 (7.00, 10.19)	9.81 (8.20, 11.21)	**< 0.001**
2hPG (mmol/L)	7.33 (6.28, 8.64)	6.46 (5.92, 8.10)	7.52 (6.53, 9.00)	**0.002**
FINS (μU/mL)	17.4 (11.3, 30.4)	6.7 (5.1, 9.5)	20.5 (14.8, 38.6)	**< 0.001**
1hINS (μU/mL)	140.0 (86.1, 223.0)	51.0 (39.8, 79.9)	175.0 (113.0, 253.5)	**< 0.001**
2hINS (μU/mL)	110.0 (59.8, 203.0)	45.0 (32.6, 58.7)	141.0 (82.6, 221.5)	**< 0.001**
HOMA-IR	4.18 (2.49, 7.14)	1.47 (1.10, 2.16)	4.99 (3.45, 8.61)	**< 0.001**
Matsuda index	2.23 (1.29, 3.66)	6.06 (4.83, 7.21)	1.97 (1.08, 2.62)	**< 0.001**
0hUCPCR (nmol/mmol)	0.64 (0.43, 0.93)	0.36 (0.25, 0.56)	0.68 (0.50, 1.00)	**< 0.001**
2hUCPCR (nmol/mmol)	2.08 (1.27, 3.20)	1.11 (0.75, 1.48)	2.42 (1.59, 3.50)	**< 0.001**

Bold values indicate a statistically significant association (P < 0.05).

A total of 192 patients with T2DM were enrolled, including 111 men (57.8%); the mean age was 53.4 ± 14.5 years, and the median diabetes duration was 7.0 years (IQR: 1.3-15.0). Among them, 95 patients were diagnosed with DMC and 97 did not. Accordingly, they were divided into a DMC group (n = 95) and a non-DMC group (n = 97). As shown in **Table 2**, compared with the non-DMC group, patients with DMC were older and had a longer diabetes duration, higher HbA1c, FPG, and 2hPG levels, and a higher proportion of oral hypoglycemic agent and insulin therapy (all *P* < 0.05). Conversely, patients with DMC had lower BMI, eGFR, TG, fasting C-peptide (FCP), 1-h C-peptide (1hCP), 2-h C-peptide (2hCP), 0hUCPCR, 2hUCPCR, and HOMA-β (all *P* < 0.05). No other clinical parameters differed significantly between groups (all *P* > 0.05).

**Table 2:** The baseline clinical characteristics of T2DM patients.

Variable	Overall (n = 192)	Non-DMC (n = 97)	DMC (n = 95)	*P* value
Male, n (%)	111 (57.8)	53 (54.6)	58 (61.1)	0.368
Age (years)	53.4 ± 14.5	48.7 ± 15.6	58.0 ± 11.7	**< 0.001**
Diabetic duration (years)	7.0 (1.3, 15.0)	2.0 (0.5, 6.0)	10.5 (7.5, 20.0)	**< 0.001**
Smoker, n (%)	51 (26.6)	24 (24.7)	27 (28.4)	0.564
BMI (kg/m^2^)	24.49 (22.23, 27.33)	25.40 (22.75, 29.00)	23.83 (22.04, 26.49)	**0.013**
SBP (mmHg)	131 ± 17	129 ± 17	132 ± 17	0.384
DBP (mmHg)	83 ± 11	82 ± 11	83 ± 11	0.426
eGFR (ml/min/1.73m^2^)	97.87 ± 21.76	104.49 ± 18.25	91.17 ± 23.03	**< 0.001**
UA (µmol/L)*	323 (266, 388)	337 (288, 393)	319 (241, 385)	0.137
TG (mmol/L)*	1.67 (1.17, 2.42)	1.84 (1.24, 2.99)	1.58 (1.11, 2.27)	**0.038**
TC (mmol/L)	4.28 ± 1.18	4.33 ± 1.19	4.23 ± 1.18	0.379
HDL-C (mmol/L)	1.05 (0.87, 1.27)	1.06 (0.88, 1.24)	1.04 (0.83, 1.36)	0.927
LDL-C (mmol/L)	2.47 ± 1.01	2.49 ± 1.02	2.42 ± 1.02	0.352
HbA_1c_ (%)	8.4 (7.0, 10.1)	8.1 (6.5, 10.0)	8.5 (7.5, 10.5)	**0.034**
FPG (mmol/L)	6.75 (5.87, 8.06)	6.41 (5.62, 7.32)	7.02 (6.14, 8.54)	**0.002**
1hPG (mmol/L)	13.85 (11.58, 16.34)	13.74 (11.72, 15.74)	13.87 (11.50, 17.03)	0.860
2hPG (mmol/L)	15.08 (12.08, 18.84)	13.50 (11.80, 17.38)	17.13 (13.28, 20.11)	**< 0.001**
FCP (nmol/L)	0.66 (0.34, 1.05)	0.87 (0.57, 1.18)	0.53 (0.24, 0.81)	**< 0.001**
1hCP (nmol/L)	1.49 (0.79, 2.57)	2.04 (1.24, 3.20)	0.97 (0.47, 1.69)	**< 0.001**
2hCP (nmol/L)	2.24 (1.21, 3.77)	3.19 (1.84, 4.50)	1.42 (0.65, 2.45)	**< 0.001**
HOMA-β	53.06 (24.04, 93.64)	73.86 (41.01, 121.57)	29.83 (16.19, 60.24)	**< 0.001**
0hUCPCR (nmol/mmol)	0.56 (0.31, 0.93)	0.66 (0.45, 0.97)	0.42 (0.23, 0.77)	**< 0.001**
2hUCPCR (nmol/mmol)	1.23 (0.62, 2.27)	1.88 (1.12, 2.61)	0.71 (0.37, 1.61)	**< 0.001**
Oral antidiabetic drugs therapy, n (%)	116 (60.4)	43 (44.3)	73 (76.8)	**< 0.001**
Insulin therapy, n (%)	76 (39.6)	20 (20.6)	56 (58.9)	**< 0.001**
Statin therapy, n (%)	68 (35.4)	19 (19.6)	49 (51.6)	**< 0.001**

The normally distributed numeric data were described as mean ± standard deviation, and the non-normally distributed data were expressed as median (interquartile range).

BMI, body mass index; WHtR, waist-to-height ratio; SBP, systolic blood pressure; DBP, diastolic blood pressure; eGFR, estimated glomerular filtration rate; UA, uric acid; TG, triglycerides; TC, total cholesterol; HDL-C, high-density lipoprotein cholesterol; LDL-C, low-density lipoprotein cholesterol; HbA1c, glycated hemoglobin; FPG, fasting plasma glucose; 1hPG, 1-hour plasma glucose; 2hPG, 2-h plasma glucose; FINS, fasting insulin; 1hINS, 1-h insulin; 2hINS, 2-hour insulin; FCP, fasting serum C-peptide; 1hCP, 1-h serum C-peptide; 2hCP, 2-h serum C-peptide; Matsuda index, Matsuda insulin sensitivity index; HOMA-IR, homeostasis model assessment of insulin resistance; HOMA-β, homeostasis model assessment of beta-cell function; 0hUCPCR, fasting urinary C-peptide/creatinine ratio; 2hUCPCR, 2-h postprandial urinary C-peptide/creatinine ratio.

*Complete data were available for 167 cases in non-diabetic individuals. Bold values indicate a statistically significant association (*P* < 0.05).

### 3.2 Spearman’s correlation analysis of UCPCRs with IR and β-cell indicators

Spearman correlation analysis was performed to assess the associations of UCPCR with IR indicators in non-diabetic individuals and with β-cell function indicators in T2DM patients, as UCPCR was a non-normal continuous variable. **Table 3** shows that in the non-diabetic individuals, both 0hUCPCR and 2hUCPCR showed significant positive correlations with HOMA-IR (*r* = 0.468, *r* = 0.531) (all *P* < 0.001) and significant negative correlations with the Matsuda index (*r* = −0.488, *r* = -0.636) (all *P* < 0.001). In the T2DM patients, significant positive correlations were found between 0hUCPCR, 2hUCPCR, and FCP (*r* = 0.668, *r* = 0.623), 2hCP (*r* = 0.569, *r* = 0.733), and HOMA-β (*r* = 0.582, *r* = 0.617) (all *P* < 0.001).

**Table 3:** Spearman’s correlation between UCPCR and HOMA-IR, Matsuda index, FCP, 2hCP, and HOMA-β.

Indicator	Correlation with 0hUCPCR		Correlation with 2hUCPCR	
	*r*	*P* value	*r*	*P* value
HOMA-IR	0.468	**< 0.001**	0.531	**< 0.001**
Matsuda index	-0.488	**< 0.001**	-0.636	**< 0.001**
FCP	0.668	**< 0.001**	0.623	**< 0.001**
2hCP	0.569	**< 0.001**	0.733	**< 0.001**
HOMA-β	0.582	**< 0.001**	0.617	**< 0.001**

UCPCR was analyzed for associations with IR indicators (HOMA-IR and Matsuda index) in non-diabetic individuals, and with β-cell indicators (FCP, 2hCP, and HOMA-β) in T2DM patients. Matsuda index, Matsuda insulin sensitivity index; HOMA-IR, homeostasis model assessment of insulin resistance; FCP, fasting serum C-peptide; 2hCP, 2-hour serum C-peptide; HOMA-β, homeostasis model assessment of beta-cell function.

Bold values indicate a statistically significant association (*P* < 0.05).

### 3.3 Multiple linear regression analysis of UCPCRs and Matsuda index in non-diabetic participants

**Figure 1:**
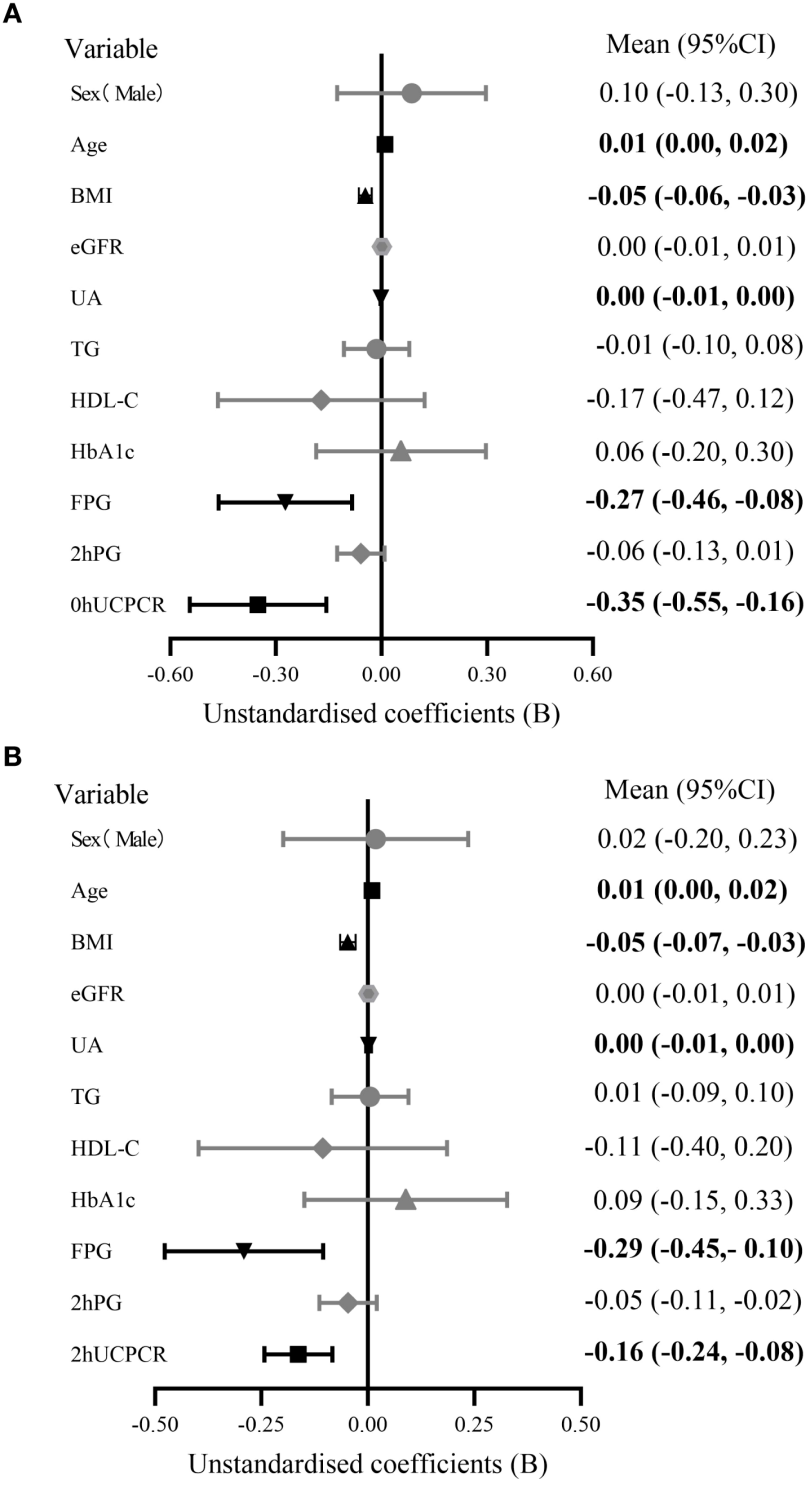
Forest plot of multiple linear regression analysis for UCPCR with the Matsuda index in nondiabetic populations. **(A)** Model incorporating 0hUCPCR and other covariates. **(B)** Model incorporating 2hUCPCR and other covariates. The analysis included 255 non-diabetic participants. Multiple linear regression was performed using enter method and all models were adjusted for sex, age, BMI, eGFR, UA, TG, HDL-C, HbA1c, FPG, and 2hPG. The Matsuda index was natural log-transformed. Data were presented as unstandardized coefficients **(B)** with 95% confidence intervals (error bars) and bold variables indicate statistical significance (P < 0.05). BMI, body mass index; eGFR, estimated glomerular filtration rate; UA, uric acid; TG, triglycerides; HDL-C, high-density lipoprotein cholesterol; HbA1c, glycated hemoglobin; FPG, fasting plasma glucose; 2hPG, 2-hour postprandial plasma glucose; UCPCR, urinary C-peptide/creatinine ratio.

**Figure 2:**
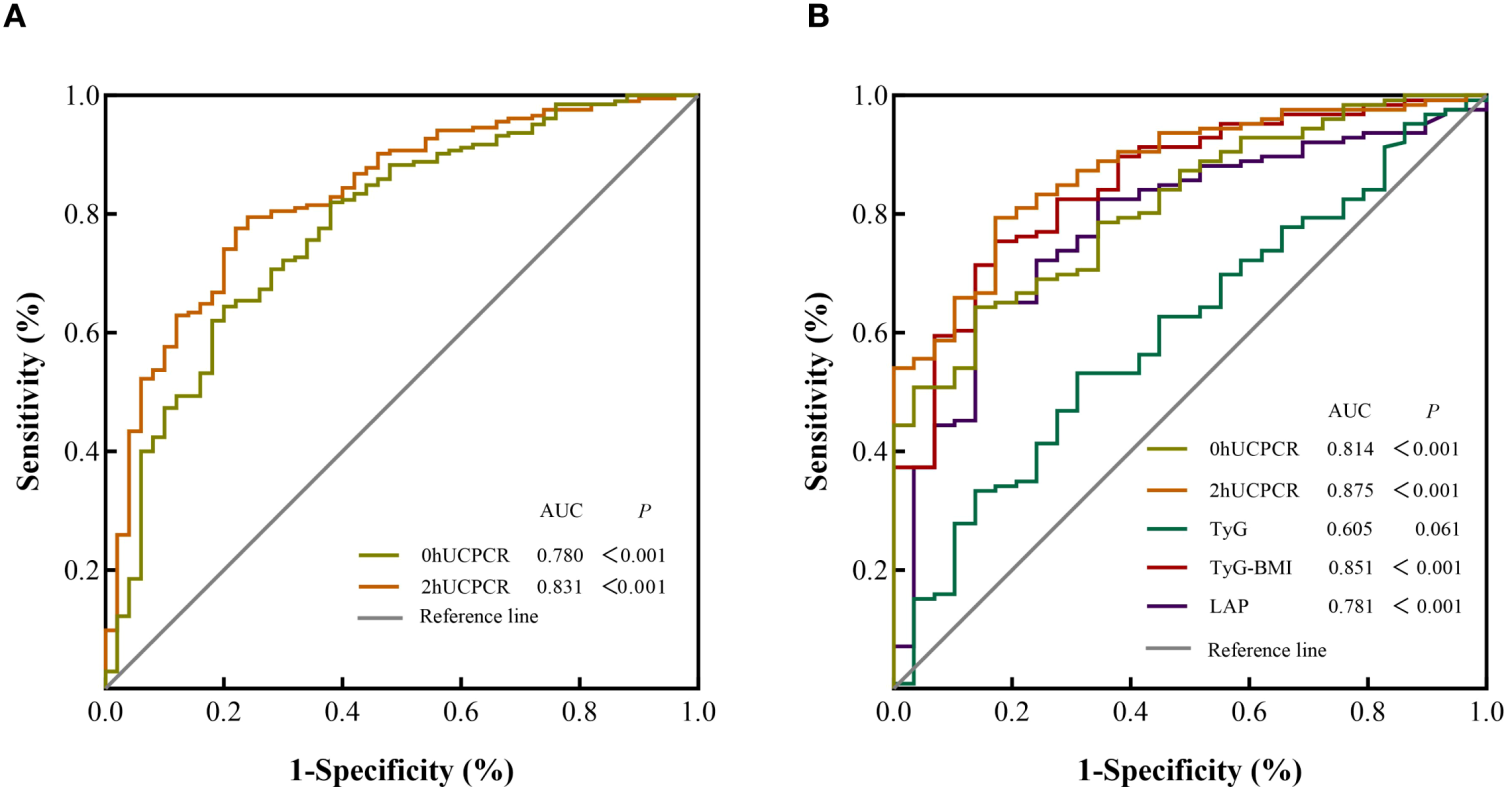
ROC curves of UCPCRs and lipid-related indices for screening insulin resistance (IR) in nondiabetic populations. **(A)** ROC curves of 0hUCPCR and 2hUCPCR for screening IR in the total non-diabetic population (n = 255). **(B)** ROC curves of 0hUCPCR, 2hUCPCR, TyG index, TyG-BMI index, and LAP index for screening IR in the subset of non-diabetic participants with complete lipid profiles (n = 167). IR was defined as Matsuda index < 4.3. The diagonal dashed line represents the reference line (AUC = 0.5). ROC, receiver operating characteristic; AUC, area under the curve; TyG, triglyceride-glucose index; TyG-BMI, TyG-body mass index; LAP, lipid accumulation product; UCPCR, urinary C-peptide/creatinine ratio.

Univariate linear regression analysis revealed that sex, age, BMI, eGFR, UA, TG, HDL-C, HbA1c, FPG, 2hPG, 0hUCPCR, and 2hUCPCR were all significantly associated with the Matsuda index (*P* < 0.05). Detailed results are presented in **Supplementary Table 1**. These variables were subsequently entered into multiple linear regression models to assess the independent associations of UCPCRs with the Matsuda index. After adjusting for sex, age, BMI, eGFR, UA, TG, HDL-C, HbA1c, FPG, and 2hPG, the results demonstrated that both 0hUCPCR (*B* = −0.351, 95% CI: −0.545, −0.157; *P* < 0.001) (**Figure 1A**) and 2hUCPCR (*B* = -0.162, 95% CI: −0.240, −0.084; *P* < 0.001) (**Figure 1B**) were independently and inversely associated with the Matsuda index. Detailed results of the multiple linear regression analysis for UCPCR with the Matsuda index are presented in **Supplementary Tables 2**, **3**.

Given that sex, glucose tolerance, and obesity status are associated with IR, subgroup analyses stratified by these three factors were performed. There were 103 men and 152 women. Glucose tolerance status was categorized into normal glucose tolerance (NGT, n = 151) and prediabetes (Pre-DM, n = 104) subgroups. Obesity status was defined as non-obese (BMI < 28 kg/m², n = 115) and obese (BMI ≥ 28 kg/m², n = 140) subgroups. Detailed results are presented in **Supplementary Table 4**. The results showed that both 0hUCPCR and 2hUCPCR were inversely associated with the Matsuda index in the NGT subgroup (all *P* < 0.001), whereas in the Pre-DM subgroup, only 2hUCPCR remained significantly associated (*P* = 0.008) (adjusted for sex, age, BMI, UA, and FPG). An inverse association with the Matsuda index was observed for both 0hUCPCR and 2hUCPCR in the obese group and the non-obese group (all *P* < 0.05) (adjusted for sex, age, BMI, UA, and FPG). An inverse association with the Matsuda index was observed for both 0hUCPCR and 2hUCPCR in the female subgroup (*P* = 0.004; *P* < 0.001), but only for 2hUCPCR in the male group (*P* < 0.001) (adjusted for age, BMI, UA and FPG).

### 3.4 Predictive value of UCPCRs for IR in non-diabetic participants

In the total population of 255 non-diabetic participants, ROC curves for 0hUCPCR and 2hUCPCR in screening IR are presented in **Figures 2A**, with corresponding AUCs of 0.780 (95% CI: 0.707, 0.853, *P* < 0.001) and 0.831 (95% CI: 0.769, 0.892, *P* < 0.001), respectively. The optimal cutoff value for 0hUCPCR was 0.58 nmol/mmol, yielding a sensitivity of 64.4%, a specificity of 80.0%, and a Youden index of 0.444. For 2hUCPCR, the optimal cutoff value was 1.51 nmol/mmol, with a sensitivity of 77.6%, a specificity of 76.0%, and a Youden index of 0.536. Detailed results are presented in **Supplementary Table 5**.

In the subset of 167 non-diabetic individuals with complete lipid data, we compared the predictive value of UCPCRs against novel IR indices (like TyG, TyG-BMI, and LAP), which extensively studied in recent years. As shown in **Figures 2B**, the analysis yielded the following AUCs: 0hUCPCR = 0.814, 2hUCPCR = 0.875, TyG-BMI = 0.851, and LAP = 0.781 (all *P* < 0.001), whereas the TyG index showed an AUC of 0.605 (*P* = 0.061).

### 3.5 Binary logistic regression analysis of UCPCRs with DMC risk in T2DM patients

Among the 192 patients with T2DM, multivariable logistic regression analyses were performed with 0hUCPCR and 2hUCPCR as independent variables and DMC as the dependent variable, with stepwise adjustment for sex, age, diabetes duration, HbA1c, and other risk factors. Four models of DMC predictors were established after adjusting for confounders (model 1: unadjusted; model 2: adjusted for sex and age; model 3: model 2 + diabetes duration, HbA1c; model 4: model 3 + BMI, eGFR, TG, history of oral antidiabetic drugs, and insulin therapy). As shown in **Table 4**, after adjusting all confounders, 2hUCPCR was independently and negatively associated with DMC risk (OR = 0.627, 95% CI: 0.411, 0.957; *P* = 0.030) and a similar inverse association for 0hUCPCR was limited to model 2 (OR = 0.387, 95% CI: 0.196, 0.764; *P* = 0.006).

**Table 4:** Logistic regression analysis of UCPCRs and DMC risk in patients with T2DM.

Model	Correlation with 0hUCPCR		Correlation with 2hUCPCR	
	OR (95%CI)	*P* value	OR (95%CI)	*P* value
Model 1	0.345 (0.172, 0.692)	**0.003**	0.424 (0.304, 0.592)	**< 0.001**
Model 2	0.387 (0.196, 0.764)	**0.006**	0.423 (0.296, 0.605)	**< 0.001**
Model 3	0.557 (0.263, 1.183)	0.128	0.581 (0.391, 0.861)	**0.007**
Model 4	0.680 (0.309, 1.497)	0.338	0.627 (0.411, 0.957)	**0.030**

model 1, unadjusted; model 2, adjusted for sex and age; model 3, adjusted for sex, age diabetes duration, and HbA1c; model 4, adjusted for sex, age diabetes duration, HbA1c, BMI, eGFR, TG, history of oral antidiabetic drugs, and insulin therapy. Bold values indicate a statistically significant association (*P* < 0.05).

Given that age, diabetes duration, and insulin therapy are associated with DMC, we performed subgroup analyses stratified by age (< 60 vs. ≥ 60 years), disease duration (< 10 vs. ≥ 10 years), and insulin therapy status (yes vs. no). In the younger subgroup (< 60 years), after adjusting for diabetes duration, HbA1c, oral hypoglycemic agent use, and insulin therapy, 2hUCPCR was significantly associated with DMC (*P* = 0.047). In the short-duration subgroup (< 10 years), after adjusting for age and HbA1c, both 0hUCPCR and 2hUCPCR were associated with DMC risk (all *P* < 0.05). In the subgroup analysis stratified by insulin therapy status, after adjusting for age, diabetes duration, and HbA1c, no independent associations were observed between either 0hUCPCR or 2hUCPCR and the risk of DMC in either subgroup (all *P* < 0.05). Detailed results are presented in **Supplementary Table 6**.

### 3.6 Predictive value of UCPCRs and integrated multivariable model for DMC in T2DM patients

**Figure 3:**
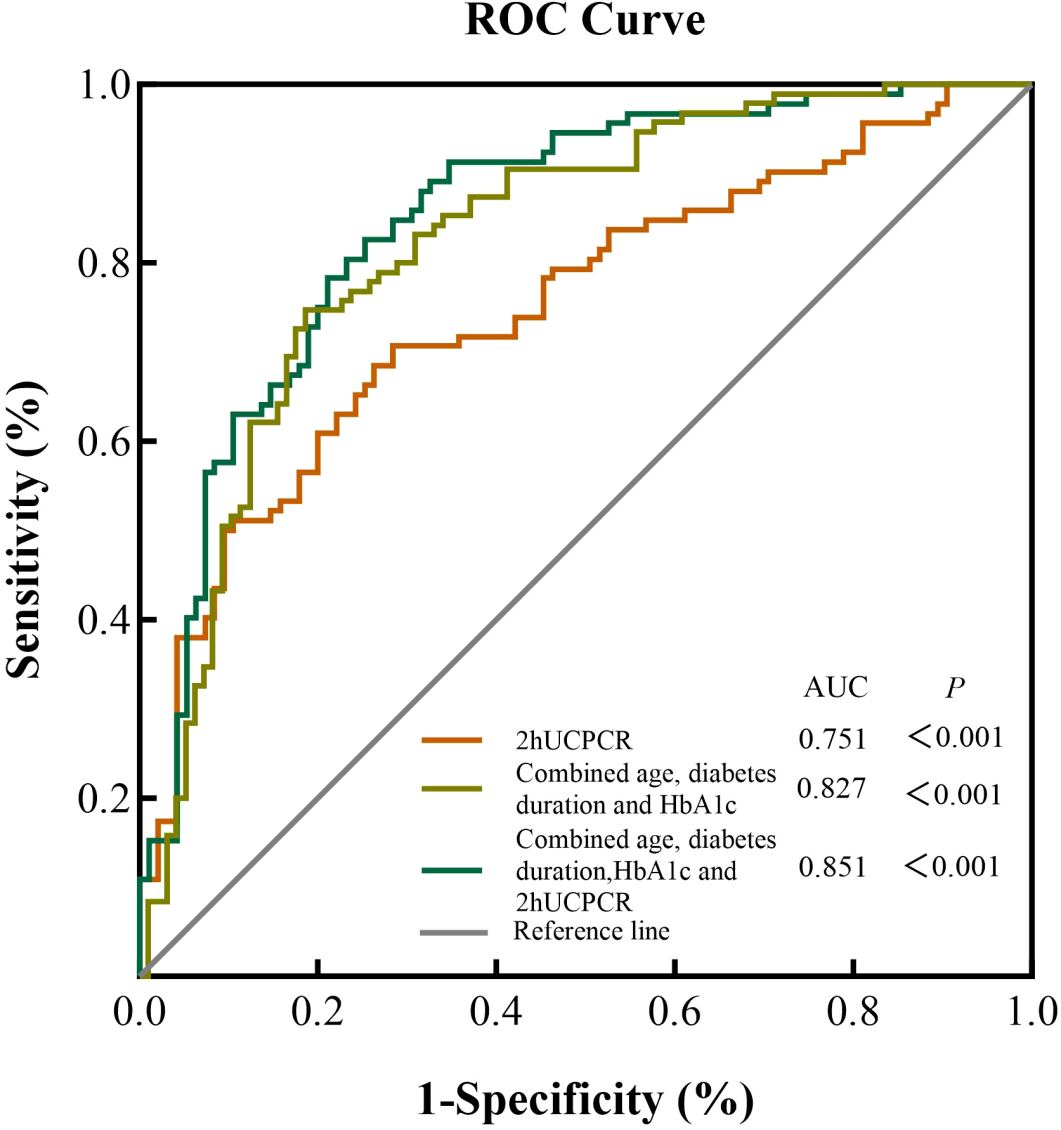
ROC curves of 2hUCPCR and other traditional risk indicators for predicting diabetic microvascular complications (DMC) in patients with T2DM. Footnotes: ROC curves comparing the predictive performance of three models for DMC risk: (1) 2hUCPCR alone; (2) the clinical model comprising age, diabetes duration, and HbA1c; and (3) the combined model integrating 2hUCPCR with age, diabetes duration, and HbA1c. The analysis included 192 patients. DMC was defined as a composite endpoint of the three microvascular complications (DKD, DR, and DPN). The diagonal dashed line represents the reference line (AUC = 0.5).

ROC curves for 2hUCPCR and other clinical risk factors (alone or in combination) in predicting DMC are presented in **Figure 3**, with corresponding AUCs of 0.751 (95% CI: 0.681, 0.821, *P* < 0.001) for 2hUCPCR alone, 0.827 (95% CI: 0.768, 0.886, *P* < 0.001) for the clinical model (age, diabetes duration, and HbA1c), and 0.851 (95% CI: 0.797, 0.907, *P* < 0.001) for the combined model (2hUCPCR combined age, diabetes duration, and HbA1c). The optimal cutoff value for 2hUCPCR was 1.22 nmol/mmol, yielding a sensitivity of 70.7%, a specificity of 71.6%, and a Youden index of 0.422. The clinical risk model achieved a sensitivity of 74.7%, a specificity of 81.4%, and a Youden index of 0.562. The combined model achieved a sensitivity of 82.6%, a specificity of 74.7%, and a Youden index of 0.573. Detailed results are presented in **Supplementary Table 7**.

### 3.7 Binary logistic regression analysis of UCPCRs with DKD, DR, and DPN risk in T2DM patients

Of the 192 patients with T2DM, 31 had DKD, 29 had DR, and 85 had DPN. Multivariable regression analyses were performed separately for each complication subtype. Following Bonferroni correction (*P* < 0.017), after adjusting for sex, age, and diabetes duration, 2hUCPCR was significantly associated with DKD (OR = 0.346, 95% CI: 0.175–0.688, *P* = 0.002), but not with DR (OR = 0.554, 95% CI: 0.314–0.979, *P* = 0.042) and DPN (OR = 0.648, 95% CI: 0.452–0.929, *P* = 0.018). No significant associations were observed for 0hUCPCR with any of the individual complications (all *P* > 0.017) (**Table 5**).

**Table 5:** Logistic regression analysis of UCPCRs and DKD, DR and DPN risk in patients with T2DM.

Subtype	Number	Correlation with 0hUCPCR		Correlation with 2hUCPCR	
		OR (95%CI)	Adjusted *P* value	OR (95%CI)	Adjusted *P* value
DKD	31	0.311 (0.097, 1.000)	0.050	0.346 (0.175, 0.688)	**0.002**
DR	29	0.438 (0.138, 1.390)	0.161	0.554 (0.314, 0.979)	0.042
DPN	85	0.621 (0.308, 1.253)	0.184	0.648 (0.452, 0.929)	0.018

All models have been adjusted for sex, age, and diabetes duration. *P* values for DKD, DR, and DPN were adjusted for multiple comparisons using Bonferroni correction; bold values indicate a statistically significant association (*P* < 0.017).

## 4 Discussion

IR and DMC represent sequential stages in the progression of T2DM. As a simple and stable surrogate marker of insulin secretion, UCPCR should be further investigated for its relationship with IR and DMC. In the non-diabetic cohort, the IR group was younger but had higher UCPCRs and a more adverse metabolic profile than the non-IR group. This included elevated lipid/glucose levels and higher rates of overweight/obesity. This observation may reflect steeper weight gain in younger populations, prompting earlier medical consultation (28). In contrast, T2DM patients with DMC were older, had longer diabetes duration, and received more intensive treatment. These patients exhibited worse islet reserve (lower C-peptide/UCPCR levels) but lower BMI and TG levels than the non-DMC group. It is likely due to sarcopenia, disease-associated frailty, and strict lipid management in advanced disease, as also noted by Li et al (29). Simple linear correlation analysis revealed that 0hUCPCR and 2hUCPCR were associated with more severe IR in non-diabetic adults and with better islet functional reserve in T2DM patients. These findings align with previous reports: Oram et al. demonstrated significant positive correlations between both UCPCR measures and HOMA-IR in 37 normoglycemic UK adults (30); Zhou et al. found that 0hUCPCR correlated positively with FCP in T2DM patients across different renal function strata (18).

In multivariable linear regression analyses of the non-diabetic population, both 0hUCPCR and 2hUCPCR remained significantly negatively associated with the Matsuda index after comprehensive adjustment, supporting their role as positive indicators of IR severity. Among T2DM patients, 2hUCPCR was independently associated with lower DMC risk after full adjustment, whereas no significant association was observed for 0hUCPCR. Compared with previous studies, Qiao et al. found that in 75 patients with T1DM, lower fasting UCPCR was associated with an increased prevalence of DR and higher cardiovascular disease risk (31). Conversely, Ji et al. demonstrated in 1,299 T2DM patients from China that fasting UCPCR positively correlated with HOMA-IR and higher DKD risk (32). The Ji cohort exhibited higher 0hUCPCR levels than ours (0.92 vs. 0.56 nmol/mmol), suggesting better preserved islet reserve and an earlier disease stage, a profile often accompanied by significant IR. Thus, elevated UCPCR in that context likely reflects compensatory hyperinsulinemia driven by severe IR rather than purely beneficial secretory capacity (19). This interpretation aligns with our observation that higher UCPCR indicated more severe IR yet predicted lower DMC risk in T2DM patients. The apparent discrepancy reflects the dual, stage-dependent role of UCPCR: Elevated levels primarily mirror IR in non-diabetic or early diabetic stages but signify preserved β-cell function in established diabetes. In non-diabetic individuals, IR is the primary driver of metabolic dysfunction. To maintain normal glycemia, pancreatic β-cells compensate by increasing insulin secretion, leading to compensatory hyperinsulinemia as reflected by elevated UCPCR levels. This finding aligns with previous studies validating UCPCR as an IR marker in obese youth (20) and in non-diabetic adults (30). As T2DM progresses, β-cell function declines due to glucotoxicity, lipotoxicity, and oxidative stress. Patients maintaining higher UCPCR levels in this advanced stage retain better endogenous insulin secretory capacity, conferring improved glycemic control and C-peptide-mediated vascular protection. The negative association between 2hUCPCR and DMC may be partly explained by the vascular protective properties of C-peptide, given that oxidative stress, inflammation, and fibrosis are key pathological mechanisms of diabetic microangiopathy (33). Experimental studies by Jeon et al. found that C-peptide supplementation attenuated vascular dysfunctions in the retina and glomerulus of diabetic mice (34). In diabetic retinopathy mice, Moon et al. reported intraocular K9-C-peptide attenuated retinal neovascularization by normalizing hyperglycemia-induced oxidative stress, vascular leakage, and inflammation (35). Mechanistically, C-peptide activates AMPK and inhibits Transglutaminase 2, which in turn suppresses reactive oxygen species (ROS) generation in endothelial cells. This reduction in ROS thereby attenuates its downstream detrimental effects, such as the upregulation of pro-inflammatory genes, enhanced vascular permeability, and endothelial cell apoptosis (33, 35, 36). These experimental findings are corroborated by clinical studies showing a negative correlation between serum C−peptide levels and DMC risk in T2DM patients, including delayed renal function deterioration with higher 2hCP levels and significantly lower fasting and postprandial C−peptide in patients with neuropathy (37, 38).

The mTOR signaling pathway, which acts as a double-edged sword in diabetes (39), may provide a mechanistic basis for the stage-dependent dual role of UCPCR observed in our study. In the early/non-diabetic stage characterized by IR, mTORC1 is activated in pancreatic β-cells as a compensatory response. Fan et al. demonstrated that amino acids promote the stability and nuclear localization of Pdx1 via the Rab1A–mTORC1 complex. Pdx1 is a master transcription factor that controls β-cell growth, function, and identity (40). A recent study by the same group further revealed that mTORC1 directly phosphorylates PDX1 at serine 61 (S61), enhancing its stability and transcriptional activity (41). This phosphorylation promotes insulin expression and β-cell proliferation, driving compensatory hyperinsulinemia. Consequently, elevated UCPCR in this stage reflects mTORC1–PDX1-mediated compensatory burden, explaining its positive correlation with IR severity. As the disease progresses to established T2DM, chronic metabolic stress leads to dysregulated mTOR signaling through two interrelated mechanisms. First, impaired mTORC1–PDX1 signaling occurs: Zhang et al. demonstrated that downregulation of Rab1A disrupts amino acid sensing via the Rab1A–mTORC1–Pdx1 axis, with Rab1A expression significantly reduced in β cells of T2DM patients correlating with loss of insulin expression (42). Additionally, genetic or acquired defects in PDX1 S61 phosphorylation, as reported by Fan et al., compromise insulin synthesis and β-cell identity (41). Second, dysregulated mTORC1 hyperactivation becomes maladaptive, with sustained signaling triggering endoplasmic reticulum stress and apoptosis that contribute to β-cell exhaustion (39). Patients who maintain higher UCPCR levels in this advanced stage may retain balanced mTOR signaling, preserving mTORC1–PDX1 axis integrity while avoiding detrimental hyperactivation. Zhao et al. further revealed that nuclear mTOR orchestrates transcriptional programs underlying cellular growth and metabolism (43), providing a mechanistic basis for how balanced mTOR signaling supports residual β-cell function and C-peptide production. These findings indicate that UCPCR reflects both early compensatory burden and late functional reserve through its link to mTOR-mediated β-cell adaptation and survival pathways.

In non-diabetic populations, 2hUCPCR showed superior performance (AUC = 0.831) compared with 0hUCPCR (AUC = 0.780) for screening IR. In recent years, accumulating evidence has confirmed that lipid-related indices such as the TyG index, TyG-BMI index, and LAP can serve as viable alternatives for IR screening. In this study, UCPCRs exhibited a certain advantage in prediction comparing with these indices. Moreover, 2hUCPCR also outperformed previously reported AUCs for TyG (AUC = 0.723, 0.744, 0.800) (44–46), TyG-BMI (AUC = 0.823, 0.729) (44, 47), and LAP (AUC = 0.796, 0.708, 0.806) (44, 46, 47) from large population-based studies including NHANES (n = 11378 adults), CHARLS (n = 10333 non-diabetic adults aged ≥ 45), and two Chinese cohorts. In T2DM patients, incorporating 2hUCPCR into a model with established risk factors (age, HbA1c, diabetes duration) increased the AUC from 0.827 to 0.851, suggesting its supplementary predictive value for DMC (48). The superior performance of 2hUCPCR over 0hUCPCR in both IR screening and DMC risk prediction stems from its role as a dynamic marker of β-cell responsiveness. This is because postprandial measures are more closely to approximate maximal secretory capacity than static fasting levels (49). Consequently, 2hUCPCR captures early defects in glucose homeostasis, which reflects systemic insulin sensitivity in the early insulin-resistant stage. As islet function declines, it signals the postprandial hyperglycemia and glycemic variability which are key drivers of DMC. Thus, it serves as a sensitive, integrated indicator of the metabolic dysfunction that precedes and promotes DMC (50). Research from Zheng et al. demonstrated that 2hCP has higher independent predictive value for the risk of DR and DPN in T2DM patients than FCP (51). This view is further corroborated by Iwamoto et al., who found that the postprandial 2hCP index was an independent predictor of successful insulin discontinuation in T2DM and was superior to the FCP index in assessing endogenous insulin secretory capacity (52).

To our knowledge, this study is the first to integrate both fasting and postprandial UCPCR measurements, establishing their screening value for IR in non-diabetic adults, and it proposes the predictive utility of 2hUCPCR for DMC risk in patients with T2DM. Moreover, in contrast to previous studies that assessed UCPCR after regular or mixed meals, our study utilized the OGTT or a standardized steamed bun meal for assessment, which significantly reduces the variability and provides support for future diagnostic criteria. However, several limitations of this study must be acknowledged. Firstly, as a cross-sectional investigation with a limited sample size, it necessitates future prospective studies with larger cohorts to confirm a causal link. Secondly, we excluded T2DM patients with CKD stage > 3a due to the impact of severely impaired renal function on C-peptide clearance, thus potentially limiting the generalizability of our conclusions to this population. Thirdly, the hospital-based recruitment limits the generalizability of our findings, and the lack of depression assessment in hospitalized T2DM patients may have introduced unmeasured confounding, as depression is known to influence diabetes prognosis. Additionally, the absence of detailed data on specific drug classes (e.g., ACE inhibitors/ARBs, GLP-1 receptor agonists, and SGLT2 inhibitors) represents a limitation. Future research with comprehensive medication records and depression evaluation is warranted. To establish causality and facilitate clinical translation, large-scale, community-based prospective cohorts are needed to confirm the relationships among UCPCR, IR, and DMC onset.

In conclusion, this study establishes a dual utility for UCPCR: elevated fasting and postprandial levels serve as accessible markers for identifying IR in non-diabetic adults, whereas decreased 2hUCPCR emerges as an independent predictor of DMC risk in T2DM patients. With their practical advantages of simplicity, stability, and non-invasiveness, UCPCRs represent promising tools for broad screening in clinical and public health settings.

## Data Availability

The raw data supporting the conclusions of this article will be made available by the authors, without undue reservation.
