# Supplementary material for: The dual role of urinary C-peptide/creatinine ratio: predicting insulin resistance in non-diabetic adults and microvascular complications risk in patients with type 2 diabetes
Source: Front Endocrinol (Lausanne). 2026 Mar 19;17:1786731. doi: 10.3389/fendo.2026.1786731 (PMC13043426; doi:10.3389/fendo.2026.1786731)
Supplement: Supplementary file 1 [file Table1.docx]

Supplementary Material

# Supplementary Figures and Tables

## Supplementary Tables

**Table S1.** Univariate linear regression analysis of UCPCRs and other independent variables with the Matsuda index in non-diabetic participants

| Variable | unstandardized coefficients (B) | 95% CI | *P* value |
| --- | --- | --- | --- |
| Sex (Male = 1) | -0.373 | -0.557, -0.190 | **< 0.001** |
| Age | 0.022 | 0.015, 0.029 | **< 0.001** |
| BMI | -0.081 | -0.092, -0.069 | **< 0.001** |
| WHtR | -5.120 | -6.020, -4.220 | **< 0.001** |
| eGFR | -0.010 | -0.017, -0.002 | **0.012** |
| UA | -0.004 | -0.005, -0.003 | **< 0.001** |
| TG | -0.112 | -0.214, -0.009 | **0.032** |
| HDL-C | 0.624 | 0.282, 0.967 | **< 0.001** |
| HbA1c | -0.560 | -0.768, -0.352 | **< 0.001** |
| FPG | -0.486 | -0.641, -0.332 | **< 0.001** |
| 1hPG | -0.126 | -0.163, -0.089 | **< 0.001** |
| 2hPG | -0.161 | -0.212, -0.110 | **< 0.001** |
| 0hUCPCR | -0.730 | -0.906, -0.553 | **< 0.001** |
| 2hUCPCR | -0.313 | -0.367, -0.259 | **< 0.001** |

Footnotes: The Matsuda index was natural log-transformed. Data were presented as unstandardized coefficients (*B*) with 95% confidence intervals (error bars). Abbreviations: BMI, body mass index; eGFR, estimated glomerular filtration rate; UA, uric acid; TG, triglycerides; HDL-C, high-density lipoprotein cholesterol; HbA1c, glycated hemoglobin; FPG, fasting plasma glucose; 2hPG, 2-hour postprandial plasma glucose; UCPCR, urinary C-peptide/creatinine ratio. Bold values indicate a statistically significant association (*P* < 0.05).

**Table S2.** Multiple linear regression analysis of 0hUCPCR with the Matsuda index in non-diabetic participants

| Variables | Unstandardised coefficients | | Standardised coefficients |  |  | 95 per cent confidence interval for B-value | |
| --- | --- | --- | --- | --- | --- | --- | --- |
|  | B | Standard error | Beta | t | Significance | Lower limit | Upper limit |
| Constant | 4.287 | 0.781 |  | 5.492 | < 0.001 | 2.739 | 5.835 |
| Sex | 0.085 | 0.107 | 0.057 | 0.801 | 0.425 | -0.126 | 0.297 |
| Age | 0.010 | 0.005 | 0.166 | 2.022 | **0.046** | 0.000 | 0.019 |
| BMI | -0.046 | 0.009 | -0.386 | -5.034 | **< 0.001** | -0.064 | -0.028 |
| eGFR | 0.001 | 0.003 | 0.032 | 0.405 | 0.686 | -0.005 | 0.008 |
| UA | -0.002 | 0.001 | -0.262 | -3.313 | **0.001** | -0.003 | -0.001 |
| TG | -0.014 | 0.047 | -0.019 | -0.293 | 0.770 | -0.107 | 0.079 |
| HDL-C | -0.172 | 0.148 | -0.075 | -1.159 | 0.249 | -0.465 | 0.122 |
| HbA1c | 0.055 | 0.121 | 0.036 | 0.451 | 0.653 | -0.186 | 0.296 |
| FPG | -0.273 | 0.095 | -0.208 | -2.865 | **0.005** | -0.463 | -0.084 |
| 2hPG | -0.059 | 0.034 | -0.135 | -1.716 | 0.089 | -0.126 | 0.009 |
| 0hUCPCR | -0.351 | 0.098 | -0.237 | -3.592 | **< 0.001** | -0.545 | -0.157 |

**Table S3.** Multiple linear regression analysis of 2hUCPCR with the Matsuda index in non-diabetic participants

| Variables | Unstandardised coefficients | | Standardised coefficients |  |  | 95 per cent confidence interval for B-value | |
| --- | --- | --- | --- | --- | --- | --- | --- |
|  | B | Standard error | Beta | t | Significance | Lower limit | Upper limit |
| Constant | 3.927 | 0.777 |  | 5.051 | < 0.001 | 2.385 | 5.468 |
| Sex | 0.016 | 0.108 | 0.011 | 0.149 | 0.882 | -0.198 | 0.230 |
| Age | 0.010 | 0.005 | 0.170 | 2.103 | **0.038** | 0.001 | 0.019 |
| BMI | -0.047 | 0.009 | -0.392 | -5.283 | **< 0.001** | -0.065 | -0.029 |
| eGFR | 0.001 | 0.003 | 0.032 | 0.419 | 0.676 | -0.005 | 0.008 |
| UA | -0.001 | 0.001 | -0.178 | -2.166 | **0.033** | -0.002 | 0.000 |
| TG | 0.005 | 0.045 | 0.007 | 0.113 | 0.910 | -0.085 | 0.095 |
| HDL-C | -0.106 | 0.147 | -0.046 | -0.719 | 0.474 | -0.398 | 0.186 |
| HbA1c | 0.089 | 0.120 | 0.058 | 0.742 | 0.460 | -0.149 | 0.327 |
| FPG | -0.290 | 0.094 | -0.221 | -3.085 | **0.003** | -0.476 | -0.104 |
| 2hPG | -0.047 | 0.034 | -0.107 | -1.369 | 0.174 | -0.114 | 0.021 |
| 2hUCPCR | -0.162 | 0.039 | -0.296 | -4.122 | **< 0.001** | -0.240 | -0.084 |

Footnotes: data are unstandardized coefficients (B) with standard error (SE), standardized coefficients (Beta), t value, and P value from multivariable linear regression analysis using the enter method. The models were adjusted for sex, age, BMI, eGFR, UA, TG, HDL-C, HbA1c, FPG, and 2hPG. The analysis included 255 non-diabetic participants. The Matsuda index was natural log-transformed before being entered into the regression model. Bold indicates statistical significance (*P* < 0.05).

**Table S4.** Multiple linear analysis of UCPCRs and Matsuda index stratified by glycemic status, BMI and sex in non-diabetic participants

|  |  | |  | 0hUCPCR | | |  | 2hUCPCR | |
| --- | --- | --- | --- | --- | --- | --- | --- | --- | --- |
| Subgroup | Number | Events, n (%) | | | *B* (95%CI) | *P* value |  | *B* (95%CI) | *P* value |
| NGT | 151 | 113 (74.8) | | | -0.447 (-0.670, -0.223) | **< 0.001** |  | -0.228 (-0.307, -0.150) | **< 0.001** |
| Pre-DM | 104 | 92 (88.5) | | | -0.120 (-0.381, 0.142) | 0.364 |  | -0.140 (-0.242, -0.038) | **0.008** |
| Obese | 140 | 137 (97.9) | | | -0.264 (-0.490, -0.039) | **0.022** |  | -0.184 (-0.253, -0.114) | **< 0.001** |
| Non-obese | 115 | 68 (59.1) | | | -0.294 (-0.546, -0.042) | **0.023** |  | -0.202 (-0.306, -0.099) | **< 0.001** |
| Female | 152 | 115 (75.7) | | | -0.308 (-0.517, -0.099) | **0.004** |  | -0.202 (-0.288, -0.116) | **< 0.001** |
| Male | 103 | 90 (87.4) | | | -0.190 (-0.463, 0.083) | 0.169 |  | -0.168 (-0.251, -0.084) | **< 0.001** |

Footnotes: NGT: normal glucose tolerance; Pre-DM: Prediabetes, includes impaired fasting glucose (IFG) and impaired glucose tolerance (IGT). Obese: BMI ≥ 28 kg/m²; Non-obese: BMI < 28 kg/m². Event number represented the number of individuals with IR in each subgroup. In the sex subgroup analysis, the model was adjusted for age, BMI, UA, and FPG. In the glucose tolerance and obesity subgroups, the models were adjusted for sex, age, BMI, UA, and FPG. Bold values indicate a statistically significant association (*P* < 0.05).

**Table S5** ROC curve analysis of 0hUCPCR and 2hUCPCR for screening insulin resistance in the total non-diabetic population

| Indicator | AUC (95%CI) | *P* value | Optimal cut-off value (nmol/mmol) | Sensitivity (%) | Specificity (%) | Youden index |
| --- | --- | --- | --- | --- | --- | --- |
| 0hUCPCR | 0.780 (0.707, 0.853) | **< 0.001** | 0.58 | 64.4 | 80.0 | 0.444 |
| 2hUCPCR | 0.831 (0.769, 0.892) | **< 0.001** | 1.51 | 77.6 | 76.0 | 0.536 |

Footnotes: Data are derived from ROC curve analysis. Optimal cut-off values were determined by maximizing the Youden index (sensitivity + specificity – 1). Bold values indicate a statistically significant association (*P* < 0.05).

**Table S6** Logistic regression analysis of UCPCRs and DMC stratified by age, diabetes duration and insulin therapy in patients with T2DM

|  |  |  | |  | 0hUCPCR | |  | 2hUCPCR | |
| --- | --- | --- | --- | --- | --- | --- | --- | --- | --- |
| Subgroup | | Number | Events, n(%) | | OR (95%CI) | *P* value |  | OR (95%CI) | *P* value |
| Age | < 60 y | 121 | 48 (39.7) | | 0.337 (0.089, 1.272) | 0.108 |  | 0.559 (0.315, 0.993) | **0.047** |
|  | ≥ 60 y | 71 | 47 (66.2) | | 0.922 (0.329, 2.581) | 0.877 |  | 0.703 (0.390, 1.269) | 0.242 |
| Diabetes duration | < 10 y | 108 | 29 (26.9) | | 0.229 (0.063, 0.832) | **0.025** |  | 0.439 (0.258, 0.747) | **0.002** |
|  | ≥ 10 y | 84 | 66 (78.6) | | 0.654 (0.249, 1.717) | 0.388 |  | 0.573 (0.318, 1.033) | 0.064 |
| Insulin therapy | yes | 76 | 56 (73.7) | | 0.777 (0.254, 2.375) | 0.658 |  | 0.587 (0.321, 1.077) | 0.085 |
|  | no | 116 | 39 (33.6) | | 0.476 (0.158, 1.434) | 0.187 |  | 0.624 (0.364, 1.068) | 0.085 |

Footnotes: Data are presented as adjusted odds ratios (OR) with 95% confidence intervals (CI). Models were adjusted as follows: for age subgroups, adjusted for diabetes duration, HbA1c, oral hypoglycemic agent use, and insulin therapy; for diabetes duration subgroups, adjusted for age and HbA1c; for insulin therapy subgroups, adjusted for age, diabetes duration, and HbA1c. The number of events represents patients with DMC in each subgroup.

**Table S7** ROC curve analysis of 2hUCPCR and other clinical risk factors for predicting DMC in patients with T2DM

| Indicator | AUC (95%CI) | *P* value | Optimal cut-off value (nmol/mmol) | Sensitivity (%) | Specificity (%) | Youden index |
| --- | --- | --- | --- | --- | --- | --- |
| 2hUCPCR | 0.751 (0.681, 0.821) | **< 0.001** | 1.22 | 70.7 | 71.6 | 0.422 |
| Combined age, diabetes duration, HbA1c | 0.827 (0.768, 0.886) | **< 0.001** | / | 74.7 | 81.4 | 0.562 |
| Combined age, diabetes duration, HbA1c, 2hUCPCR | 0.851 (0.797, 0.907) | **< 0.001** | / | 82.6 | 74.7 | 0.573 |

Footnotes: Data are derived from ROC curve analysis. Optimal cut-off values were determined by maximizing the Youden index (sensitivity + specificity – 1). Bold values indicate a statistically significant association (*P* < 0.05).
